# Mechanical and Unlubricated Sliding Wear Properties of Nitrile Rubber Reinforced with Micro Glass Flake

**DOI:** 10.3390/polym10070705

**Published:** 2018-06-26

**Authors:** Yanbao Guo, Hai Tan, Zhiqiang Cao, Deguo Wang, Siwei Zhang

**Affiliations:** 1 College of Mechanical and Transportation Engineering, China University of Petroleum, Beijing 102249, China; doc.tan@outlook.com (H.T.); czar2003@126.com (Z.C.); swzhang99@sina.com (S.Z.); 2 Beijing Key Laboratory of Process Fluid Filtration and Separation, Beijing 102249, China; 3 China Petroleum Pipeline Engineering Co., Ltd., Langfang 065000, China

**Keywords:** micro glass flake, nitrile rubber, wear resistance, friction and wear

## Abstract

In this study, the filled nitrile rubber (NBR) was prepared with micro glass flake (GF). The tribological behaviors of filled NBR were tested by a ball-on-disk tribometer. Material properties such as glass transition temperature (*T_g_*), fracture energy, tensile strength and dispersity of GF filler were also investigated. The results showed that the coefficient of friction (COF) of NBR reduced and the wear-resistant enhanced with the GF filler. Compared to unfilled NBR, the COF of filled NBR suffered a maximal drop percent (about 21.1%) at a rotation speed of 100 rpm and normal load of 1.5 N. Mechanical and wear behaviors were dependent on the interfacial performance of filler in the rubber matrix. Filler with smaller size was more conducive to enhance the interfacial strength of the polymer matrix. That can increase the interfacial strength of filler and benefits to improve the anti-wear behavior of rubber.

## 1. Introduction

As a preferred material for many applications, nitrile rubber (NBR) is widely used in various industries including sealing and anti-wear components because of its good properties such as physical, chemical and thermostability [1,2,3]. Accordingly, a great deal of scientific researches have been focused on the properties of NBR and its applications. However, according to the failure statistics, the wear of rubber is one of the foremost contributors to the failure of polymer parts [4]. It was pointed out that wear of rubber was the main cause of these failures, which account for 60~80% [5]. Furthermore, the wear of rubber is still an inevitable problem for the seal components and moving components under rough contact and harsh terms, especially in a moving system. This could lead to an enormous economic loss [6]. For example, in screw pump systems, the wear in elastomer stator is a time-consuming and costly problem [7]. A lot of researches have been carried out to investigate the wear behaviors of rubber, for instance, Cao et al. [8] prepared NBR with different cross-linking densities and studied the cross-linking heterogeneity effects on the wear behaviors of NBR. It was found that the cross-linked heterogeneity shows a critical factor in wear properties of unfilled NBR. Furthermore, the operating conditions and its structure factors also influence the wear behaviors of polymer. Chen et al. [9] studied the friction and wear performances of polymers sliding against GCr15 and 316 steels under sea water lubricant condition. From the test results, it was found that the tribological properties of a polymer with the lubrication of sea water were not only influenced by the properties of the polymer itself, but also the corrosion and lubrication of the aqueous medium. Shen et al. investigated the tribological behaviors of NBR in two-body abrasion. The results indicated that the size of abrasive showed a significant effect on the tribological and mechanical properties of the NBR [10]. Guo and co-authors [11] prepared bimodal NBR and evaluated the friction and wear performances of rubber. The test results showed that the pattern of the rubber network could be transferred from the elastomeric to the thermosetting resin.

In addition, to improve the wear performance of rubber and meet the demands of rubber application, more and more polymer composites with enhanced behaviors are studied including many types of fillers-reinforced polymers [12]. It is beneficial to satisfy the urgent demands of environmental protection, energy saving, cost reducing, and emission reduction [13]. Therefore, the research of polymer composite materials with filler has been one of the most popular research fields [14]. Gatos and Karger-Kocsis [15] studied the influences of the aspect ratio of layered filler on the oxygen permeation and mechanical behaviors of hydrogenated nitrile rubber (HNBR) filled with organophilic layered silicate. Expanded graphite (EG)- and modified expanded graphite (MEG)-filled composite polymers were reported to be fabricated based upon natural rubber with or without carbon black [16]. EG/MEG added natural rubber compounds, whether in the presence of carbon black showed comprehensive enhancement, such as in the thermal properties, dynamic mechanical, cure characteristics, and so on. Furthermore, fibrillar structure fillers such as fibrillar silicate were also used to load into the polymer in order to enhance its mechanical, tribological and thermal performances [17,18]. The investigation indicated that the dispersion of the fibrillar silicate filler and interfacial strength between the rubber and fibrillar silicate were able to be improved by modifying the fibrillar silicate surface, thus the polymer’s mechanical performances were improved [19]. In the previous investigations, the tribological properties of NBR which contains hollow glass beads (HGB) showed that the coefficient of friction (COF) of NBR was reduced and reinforced wear-resistant was obtained by adding the HGB filler into the rubber matrix. Furthermore, the contact interface between the steel ball and NBR could also be improved by the chips of HGB, and the extension of curl-shaped worn of rubber surface was impeded because of the chips on the frictional interface [20].

According to the previous researches, glass flake (GF) is known as a high aspect-ratio reinforcing additive which appears in many commercial applications, such as anti-corrosion [21,22], fire protection [23], and denture materials [24]. The micro flake is a modified ‘C’ glass composition which is used as filler to improve the mechanical performances of polymer composites. For instance, the glass flake-reinforced polypropylene (PP) was prepared for analyzing the impact resistance. Results indicated that the dynamic fracture toughness of the glass flake-filled PP composite increased obviously, and it was intensely rate-dependent with the adding of glass flakes [25]. Glass flakes can substantially improve the denture base acrylic material polymethyl methacrylate (PMMA) in fracture toughness for a 69% increase [24]. Klaas et al. [26] investigated the tribological behaviors of glass-filled polytetrafluoroethylene (PTFE), and the results showed that PTFE composites filled with different glass fillers behaved in different abilities for forming transferred film on the counterface, and then affected their sliding wear. Therefore, it has attracted increasing attention in many industrial polymer fields to use glass flake as a reinforcing agent.

In this study, the NBR-vulcanized specimens were prepared with different sizes micro glass flakes in accordance with a certain formula proportion. GF were blended into nitrile rubber step by step through a two-roll mill and the sulfuration process. In order to characterize the NBR vulcanizate, universal tests were carried out including dynamic mechanics analysis (DMA) and differential scanning calorimetry (DSC). The tribological properties of GF-reinforced NBR have been examined using a ball-on-disk frictional instrument. Furthermore, the micro wear tracks were observed by scanning electron microscope (SEM) after the friction test.

## 2. Experiment Section

### 2.1. Materials and Preparation of NBR Specimens

**Figure 1:**
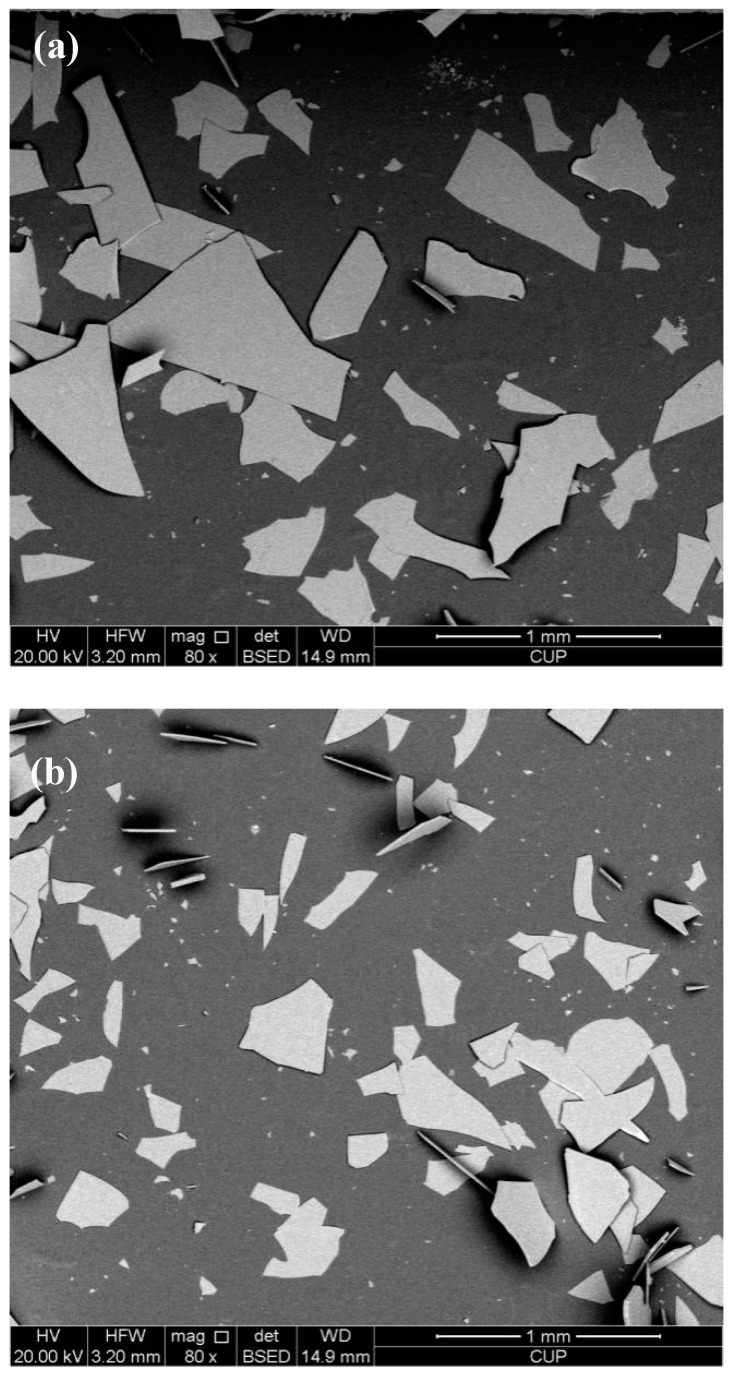

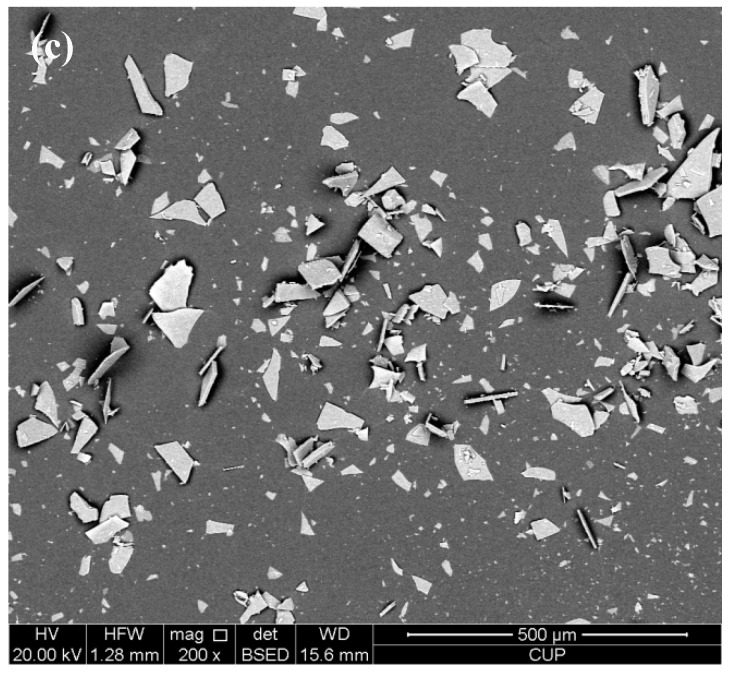
SEM images of three type micro glass flakes: (**a**) GF-B, (**b**) GF-M and (**c**) GF-S.

Nitrile rubber (N215SL, contain with 48% of acrylonitrile, JSR Corporation, Tokyo, Japan) was used as basis material. Sulfur (99.5% purity, Langfang, China) was purchased from Kezhan Chemical Company and used as the curing agent. The micro glass flakes (GF, ‘C’ glass) were supplied by Glassflake Australia Pty Ltd. (Perth, Western Australia). The chemical constituents of micro glass flake are given in Table 1. Three styles of GF with different lengths are employed as filler in this study. The micro morphology of the three styles GF with different sizes are shown in these SEM images (Figure 1). The average lengths of three styles GF are 425 um, 300 um and 35 um, respectively. The nominal thickness of the glass flake is around 5 ± 2 μm. Thereafter, the filled NBR with three GF are represented as GF-B, GF-M and GF-S for lengths of 425 μm, 300 μm and 35 μm, respectively.

Moreover, *N*-Cyclohexy-2-benzothiazole sulfonamide (CBS) was used as the vulcanization accelerator as well as tetramethyl thiuram disulfide (TMTD). Additionally, other ingredients such as zinc oxide (ZnO), stearic acid, and sulfur were commercial grade chemicals. These chemicals were used as supplied without further processing. Table 2 shows the specific formula proportion of samples. Vulcanizates of NBR were fully mixed by a two-roll mill. Afterwards, a hydraulic press was utilized to prepare the NBR samples under a pressure of 20 MPa and temperature of 155 °C. Additionally, a moving die rheometer (GoTech-M2000A, Taiwan, China) was used to identify the optimal cure time.

### 2.2. Experimental Instruments and Measurements

#### 2.2.1. Mechanical Tests

The universal test machine (WDTII-20, Guangzhou, China) was used for evaluating uniaxial tensile properties of rubber specimens. In this study, according to the standard of ISO 37: 2011, the crosshead velocity was 500 mm/min. Before the test, the two clamp holders of instrument were set with an initial space of 36 mm. Then, the average value can be obtained from results of five different samples. Furthermore, the fracture energy (*G_c_*) of specimen was measured with the trouser tear test mode which was also performed by the universal test machine. The crosshead velocity was 5 mm/min. According to Equation (1), the fracture energy, *G_c_*, of NBR can be calculated according to following equation [27].

(1) $$G_{c}=\frac{2F}{t}$$

where, *G_c_* is the fracture energy of rubber specimens. *F* is the tear force. *t* is represented as the thickness of the test piece.

#### 2.2.2. Differential Scanning Calorimetry (DSC)

DSC instrument (NETZSCH, 204F1, Bavaria, Germany) was used to evaluate the DSC characterization. The test temperature was ranged from −70 °C to 20 °C, and the rise rate was 10 °C/min. Furthermore, the glass transition temperature (*T_g_*) of specimens can be determined from the DSC curves through the mid-point of transition.

#### 2.2.3. Dynamic Mechanics Analysis (DMA)

A dynamic mechanical thermal analysis (DMA) instrument (NETZSCH, DMA242C, Bavaria, Germany) was used to conduct the DMA of NBR specimens. The DMA test was performed under the uniaxial stretch mode. Furthermore, the dynamic tensile modulus was measured in a temperature sweep test (from −60 °C to 30 °C), under the rise rate of 3 °C/min and the frequency of 1 Hz.

#### 2.2.4. Friction and Wear Tests

**Figure 2:**
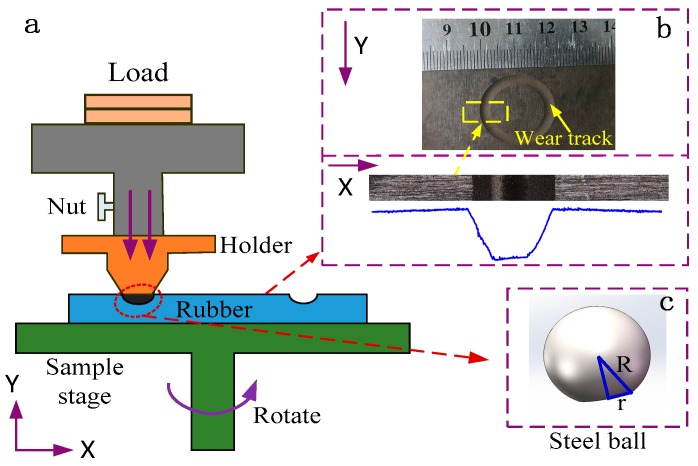
The test configuration of ball-on-plate tribometer: (**a**) equipment diagram, (**b**) wear track of NBR specimen and (**c**) wear of counterpart.

The experiments were operated using an ultra-functional tribological testing machine under a ball-on-disk mode (CFT-I, Lanzhou, China) in ambient air (open air). Figure 2 shows the test configuration of ball (steel)-on-plate (rubber) without lubricants. In this study, the GCr15 steel ball was set as the counterpart. The surface roughness and diameter of the steel ball is about 0.8 μm and 5 mm, respectively. Before the tribological test, the steel counterpart samples were cleaned in absolute ethyl alcohol, acetone and pure water with ultrasound for 10 min, respectively. Finally, the samples were dried in a stream of nitrogen. The NBR specimens were vulcanized into square with side length of 50 mm and 2 mm thick. Before the tribological test, the NBR specimens were bonded onto the steel disc for fixing. The tribological experiments were operated at the condition with a rotation speed of 100 rpm and a rotation radius of 10 mm. The applied constant normal loads were set up from 1 N to 3.5 N. The friction tests were carried out at a room temperature of 25 °C with 25% relative humidity in air. During the sliding process, the COF was measured continuously from the frictional curve by strain gauges and the wear rate (*R_w_*) of rubber sample in volume can be obtained as follows:

(2) $$R_{w}=\frac{\Delta V}{LN}$$

where Δ*V* is the wear volume loss of sample. *L* is the sliding distance during the frictional test, and *N* is the normal load. As the surface wear track shown in Figure 2b, the wear volume loss can be obtained by Equation (3).

(3) $$\Delta V=S\times 2\pi R_{t}$$

where, *R_t_* is the radius of the friction track. *S* is the cross-section area of the friction track.

Additionally, the counterpart steel ball in rubber/metal tribo-pairs may also be worn. In this study, the wear behavior of the steel ball was related to a specific value *k* = *r*/*R*, where *R* is the radius of the steel ball, and *r* is radius of the worn surface of the steel ball as shown in Figure 2c.

#### 2.2.5. Scanning Electron Microscopy (SEM)

High resolution images of the wear tracks on the surface of samples were observed by SEM (FEI Quanta200F, Hillsborough, OR, USA). Prior to the SEM measurement, all the NBR samples were coated with gold film by sputtering.

In addition, to investigate the morphology and dispersibility of GF filler in the rubber bulk, the dumbbell-shaped specimens were tested using a low temperature impact test apparatus under liquid nitrogen environment, and the images of fracture surface were also observed using SEM.

## 3. Results

### 3.1. Characterization of GF-Filled NBR

**Figure 3:**
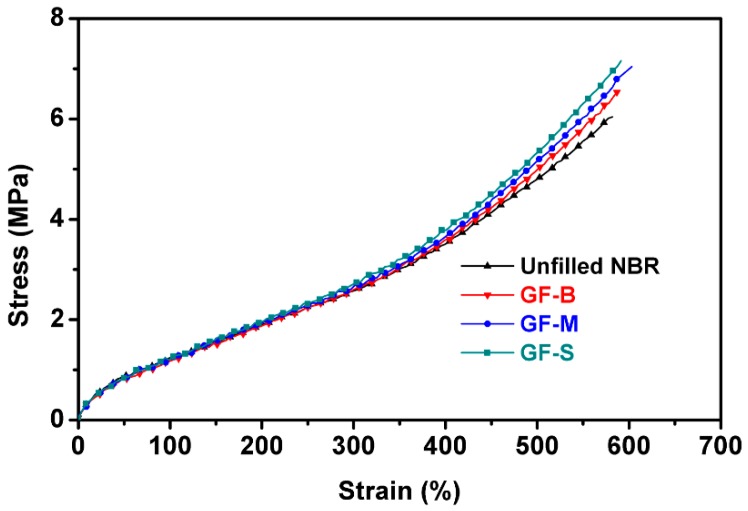
Stress-strain curves of sulphur-vulcanized NBR specimens.

Figure 3 shows group tensile test results of the filled and unfilled nitrile butadiene rubber. Five group tests under the same parameters were carried out. From the stress-strain curves of different NBR vulcanizates, the incorporation of glass flake within a rubber matrix does not have an obvious change in the tensile behavior. The average tensile strength of different NBR specimens were listed in Table 3. Compared with unfilled NBR, it can be observed that the average tensile strength increased with glass flake composite. However, the tensile strength just increased slightly with the filler size of glass flake decreasing.

**Figure 4:**
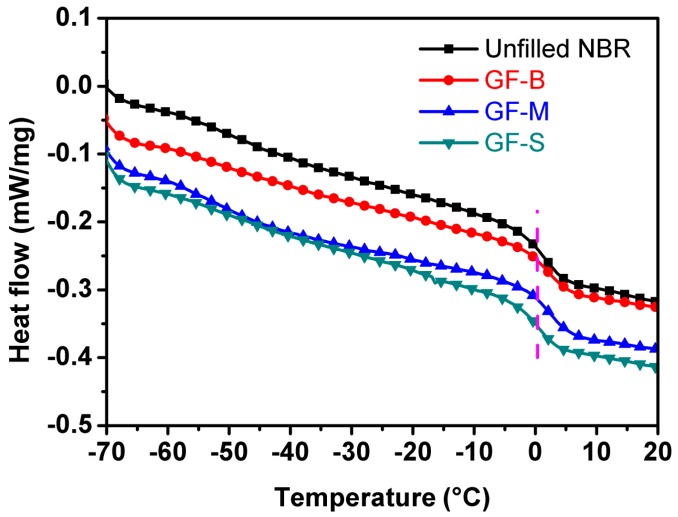
Differential Scanning Calorimetry (DSC) curves of glass flake-filled and unfilled NBR specimens.

Figure 4 displays the heat flow as functions of the temperature (−70 to 20 °C) by a DSC instrument. From the DSC curves (shown in Figure 4), it can be noted that the temperature range which is related to the mutation range of DSC baseline is consistent for each sample. Accordingly, the glass transition temperatures (*T_g_*) are around 0 °C for all the NBR specimens as shown in Figure 4. It can be deduced that the introduction of GF filler would not show a significant effect on the movement of the polymer chain segment.

It is well known that the tribological performances of materials could be influenced by the basic mechanical performances. Thus, the hardness of different NBR specimens were measured by a sclerometer (LX-A, Beijing, China) according to the ISO 7619: 2004. The hardness of different NBR vulcanizates are listed in Table 3. As shown in Table 3, the unfilled NBR behave a shore hardness (A) of 44, approximately and the shore hardness after mixing with GF is larger than the unfilled NBR. This is resulting from the influence of inorganic fillers which could improve the load-carrying capacity [28,29]. Moreover, the hardness increased a little with the decreasing of GF size. It may be corresponding with the dispersibility of fillers in the polymer bulk and the bonding strength between interfaces of GF filler and rubber.

**Figure 5:**
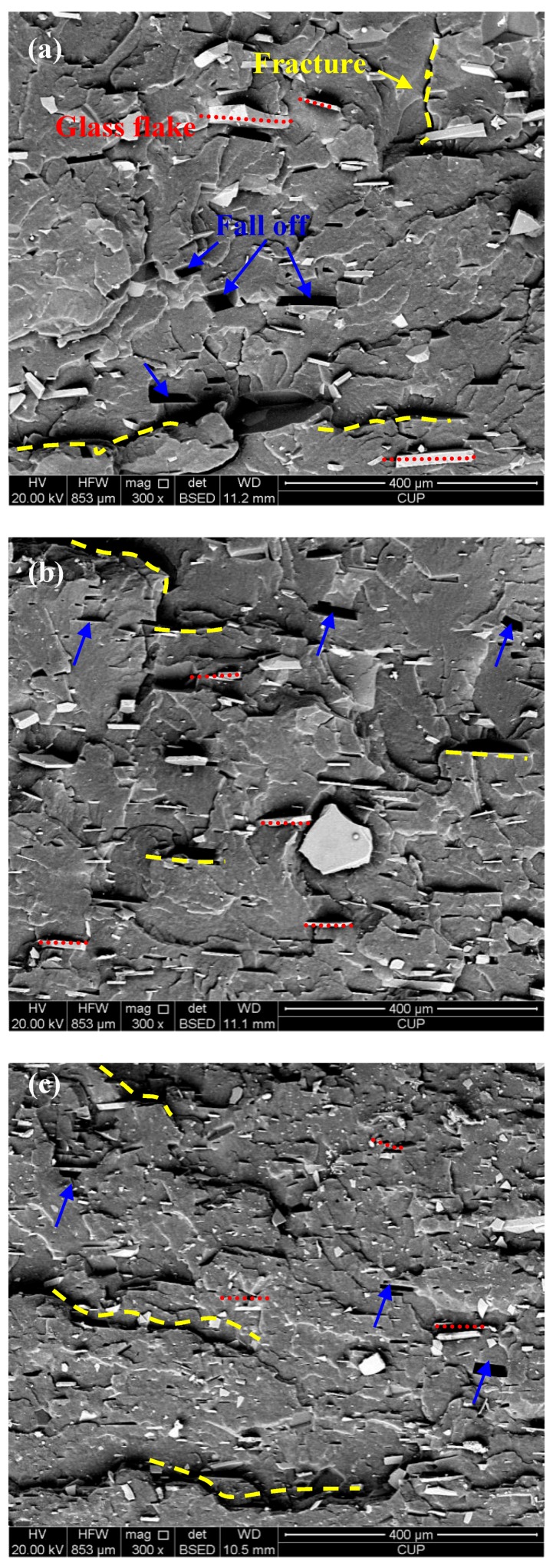
SEM images of fracture surfaces of glass flake-filled NBR: (**a**) GF-B, (**b**) GF-M and (**c**) GF-S.

In order to investigate the dispersibility of GF filler in the rubber matrix, the NBR fracture surfaces were tested under a low temperature brittle fracture method. Figure 5a–c show the SEM images of filled NBR specimens for GF-B, GF-M and GF-S, respectively. From the cross-section fracture surfaces, typically brittle and tearing fracture features can be found. From the SEM images, for the most part, the GF fillers are horizontal in the rubber structure after being cured by hydraulic vulcanizing press. Furthermore, it can be found that some GF fillers fell off on the fracture surfaces. Compared with three images as shown in Figure 5, it can be found that the bigger size GF is easier to fall off from the rubber mixture fracture surface. On the whole, the GF filler has a good uniform dispersion in the rubber matrix.

**Figure 6:**
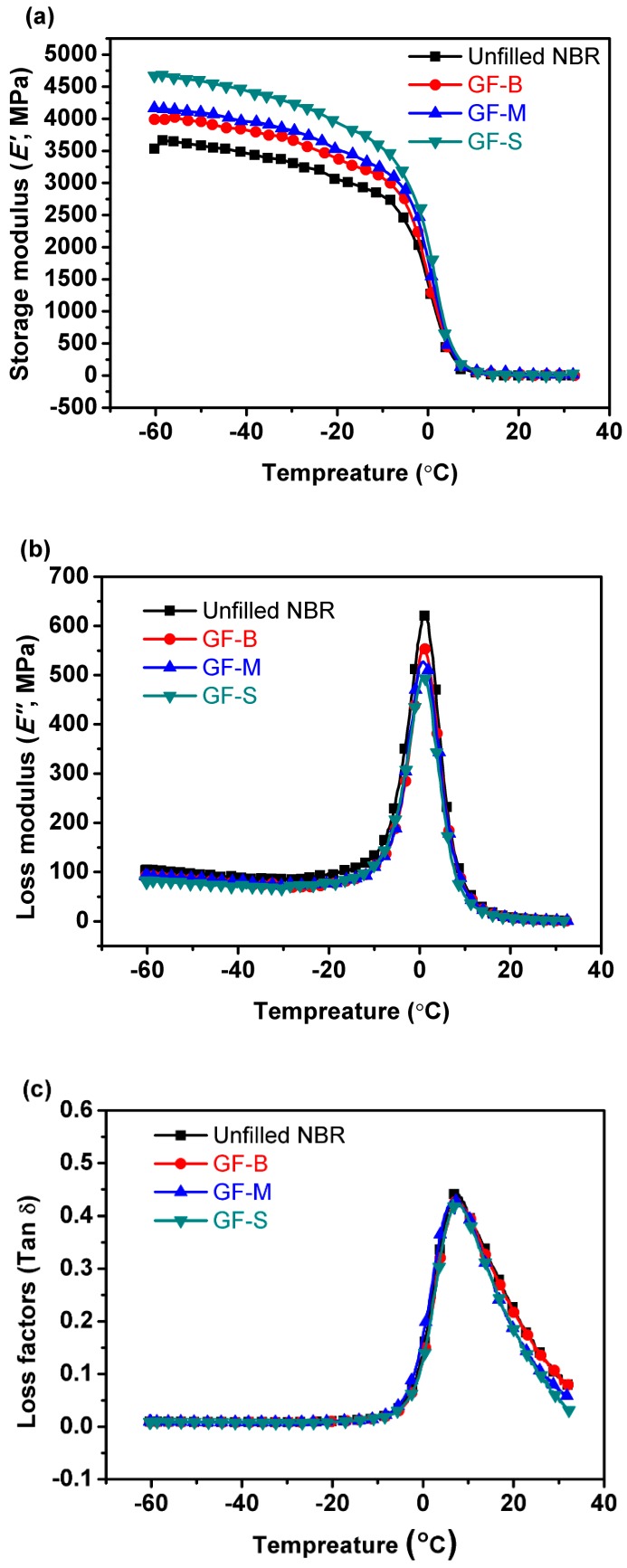
The Dynamic Mechanics Analysis (DMA) patterns of NBR vulcanizates: (**a**) storage modulus (*E*’), (**b**) loss modulus (*E*”) and (**c**) loss factor (tan δ).

Additionally, the DMA investigation of NBR vulcanizates is observed using a DMA instrument (NETZSCH, DMA242C, Bavaria, Germany). Figure 6 shows the DMA results of different NBR specimens. From the storage modulus (*E*’) curves as shown in Figure 6a, the storage modulus increased after filled with GF in the low-temperature stage and the storage modulus were almost same for the three NBR specimens with different size GF filler at 20 °C, which was about 13.5 MPa. Moreover, the elasticity modulus after filled with GF showed no obvious change than the unfilled NBR as listed in Table 3. The loss modulus and factors (*E*”, Tan δ) were shown in Figure 6b,c, respectively. With the decrease of GF size, the *E*” reduced as shown in Figure 6b. Furthermore, Tan δ shows the characteristic of one peak which can be found in Figure 6c. All of the results indicate that the networks of all GF-filled NBR samples are typically complete and continuous rather than a two-phase [30]. Additionally, the elasticity modulus of NBR is not changed obviously. What is more, the range of transition gets narrower with the decreasing sizes of GF which indicates that GF-S shows a higher miscible than GF-B and GF-M [31].

### 3.2. Tribological Properties of NBR Filled with GF

The tribological behaviors of unfilled and GF-filled NBR samples under dry condition were tested using a ball-on-disk frictional instrument, which the schematic diagram is shown in Figure 2. In the sliding test, the different NBR samples rotate together with the electromotor at a velocity of 100 rpm. The coefficient of friction was measured by the friction sensor.

**Figure 7:**
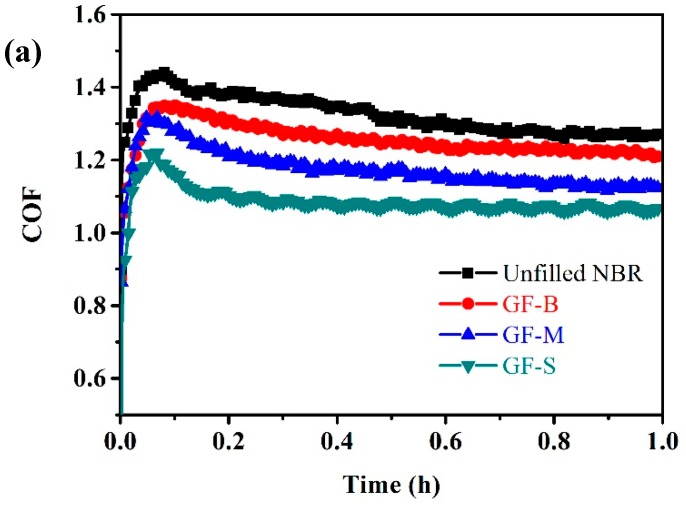

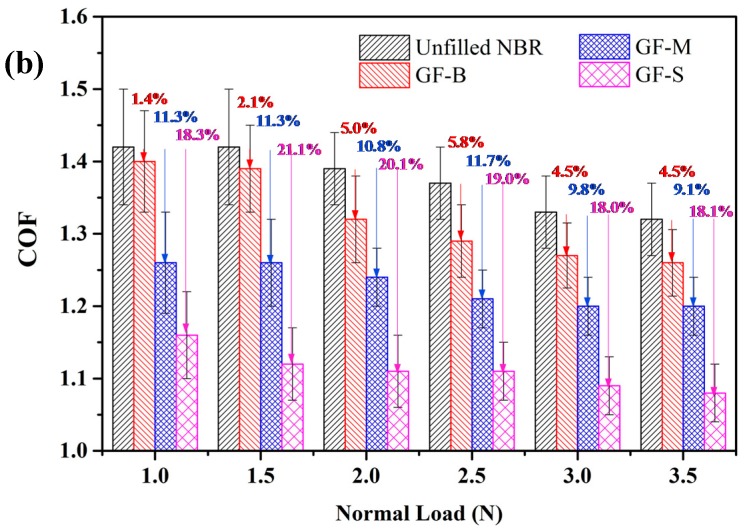
(**a**) Coefficients of friction (COF) versus friction time for filled and unfilled NBR specimens under a rotation speed of 100 rpm and normal load of 3.5 N; (**b**) COF and the declining percent of NBR specimens under different normal loads.

Figure 7a shows the typical curves of COF with respect to the rotating time under the applied normal load of 3.5 N. From the COF curves, at the inception region, the COF rose sharply at first and then dropped down. This represents a running-in process of the sliding friction. Moreover, after the steel ball sliding about 20 min through the friction running-in process, the steady-state frictional region could be observed for each NBR sample. On the whole, the COF of NBR reduces after filled with GF. Without the GF filler, the steady-state COF of NBR was fluctuating around 1.32. When with the GF filler, the COF reduces obviously, especially for GF-S NBR. The COF of GF-S-filled NBR decreased to about 1.08. Figure 7b shows the COF and the declining percent with respect to the normal loads from 1 N to 3.5 N, after 1 h friction process. It can be seen that the COF decreases with the increasing of the normal load. The heat is generated on the contacting points when friction occurs. The variation of temperature further affects the COF. When the normal load increases, the temperature between ball and NBR enhances. The elevated temperature softens the hardness of NBR and the COF declines [32,33]. The extent of decline of COF is ranging from 1.4% to 5.8% for GF-B NBR at different normal loads. The GF-S NBR suffers the lowest COF in same experimental condition compared with other filled NBR. With the increasing of normal load (from 1 N to 3.5 N), the COF of GF-S NBR changes from 1.16 to 1.08. When at a normal load of 1.5 N, the COF declines about 21.1% compared with unfilled NBR sample.

**Figure 8:**
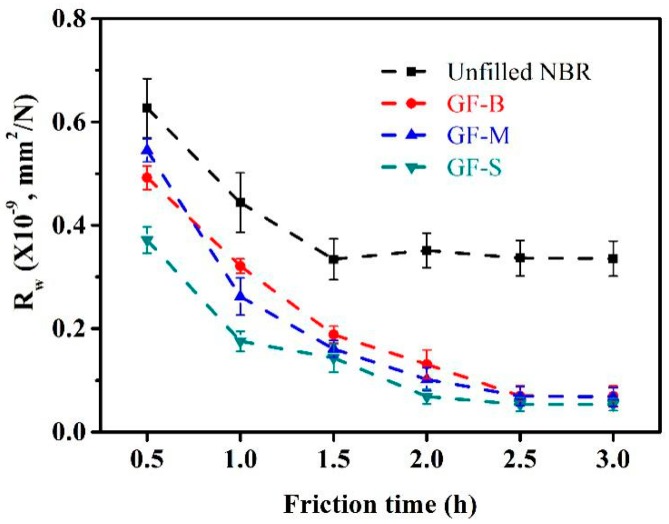
The wear ratios versus sliding time for filled and unfilled NBR specimens.

**Figure 9:**
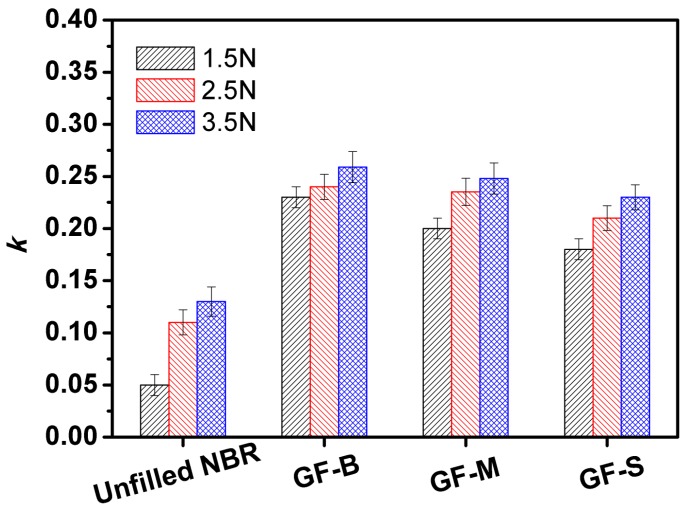
Wear ratios of counterpart steel ball against unfilled and GF-filled NBR specimens under normal load 1.5 N, 2.5 N and 3.5 N.

Additionally, the wear behaviors of NBR samples are shown in Figure 8. Compared with the unfilled NBR specimen, the wear resistance is enhanced obviously with the filler of GF. From the line charts of wear rates as shown in Figure 8, it is found that the wear loss decreased as the friction time increased. For unfilled NBR, the wear rates change from 0.63 × 10^−9^ mm^2^/N to 0.34 × 10^−9^ mm^2^/N with the friction time increasing from 0.5 h to 3 h. The GF-S NBR also has the same trend, and its wear rates drop from 0.37 × 10^−9^ mm^2^/N to 0.05 × 10^−9^ mm^2^/N. It is because of the contact region enlarged with the increase in the friction time. Thus, the contact pressure declines due to the increasing of the area at contact interfaces. Furthermore, the wear rate is also affected by the glass flakes which appear on the sliding interfaces between the steel ball and NBR specimens. Figure 9 shows the wear loss (wear ratio *k*) of the steel ball counterpart after 3 h, under varied normal loads with different GF fillers. Compared with the unfilled NBR condition, the wear ratio of steel ball shows a little increase with the filler of GF. Furthermore, the wear ratio of steel ball only increases slightly with the increase in the normal load, and the influence of filler size on the wear loss of steel ball counterpart is not obvious. It is slightly decreased with the reduction of GF filler size.

**Figure 10:**
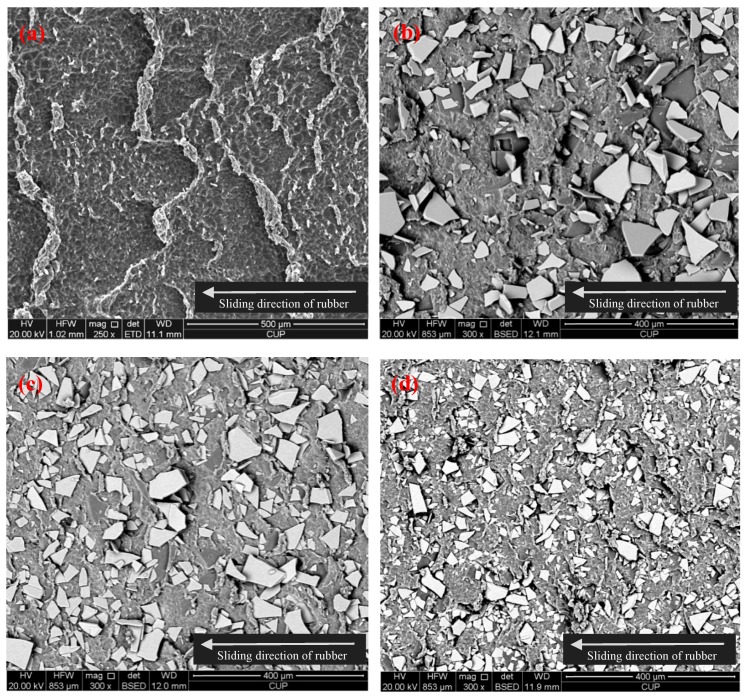
SEM images of abrasive surfaces of (**a**) unfilled NBR, (**b**) GF-B-, (**c**) GF-M-, and (**d**) GF-S-filled NBR specimens.

**Figure 11:**
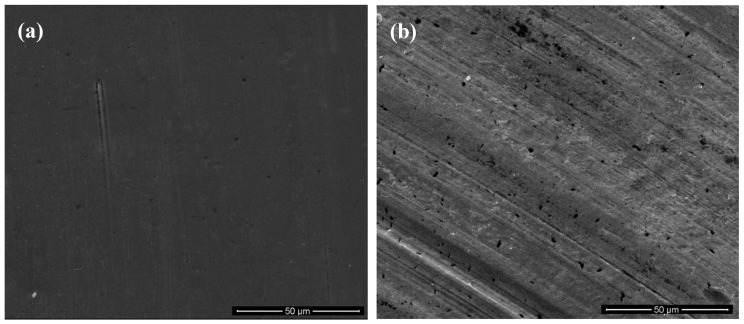
Worn surface of steel ball against (**a**) unfilled NBR and (**b**) GF-S-filled NBR specimens.

Figure 10 shows the surface wear tracks of different NBR specimens. For the unfilled NBR sample, the typical wear patterns for rubber materials are observed as shown in Figure 10a. From the abraded surface of unfilled NBR, a series of parallel ridges are generated on the wear surface, which are almost perpendicular to the sliding direction. As previous studies indicated, the periodic wear patterns of surface are a common feature in the viscoelastic materials [34,35]. The formation of the periodic wear patterns (wavy pattern) is due to a complex compression and extension strain cycle in the contact interfaces, and this is a typical wear phenomenon for special rubber-like materials [36,37,38,39]. Moreover, some curled debris can be seen on the worn surface as shown in Figure 10a. It can be deduced that the local adhesion was present between the rubber and steel surface, and then the adhesive rubber was lengthened which is repeated in the friction process. Compared with the particular appearance for the unfilled NBR, little pits and small ridges occur in the worn surfaces of GF-filled NBR specimens shown as Figure 10b–d. In summary, the COF of NBR filled with GF decreased and its anti-wear performance enhanced. Figure 11a shows the worn surface morphology of the steel ball against unfilled NBR sample. It can be found that no obvious damage appeared on the steel ball counter surface. Moreover, Figure 11b shows the damage morphology of the steel ball against the GF-S-filled NBR sample. Compared with Figure 11a, serious ploughs were observed on the steel ball surface due to the GF particles present on the contact interfaces. Therefore, the main wear mechanism of the steel ball was the abrasive wear caused by the hard glass particles embedded in the rubber, which could accelerate the wear of metal.

## 4. Discussions

### 4.1. Effect of GF Filler on the Friction of NBR

**Figure 12:**
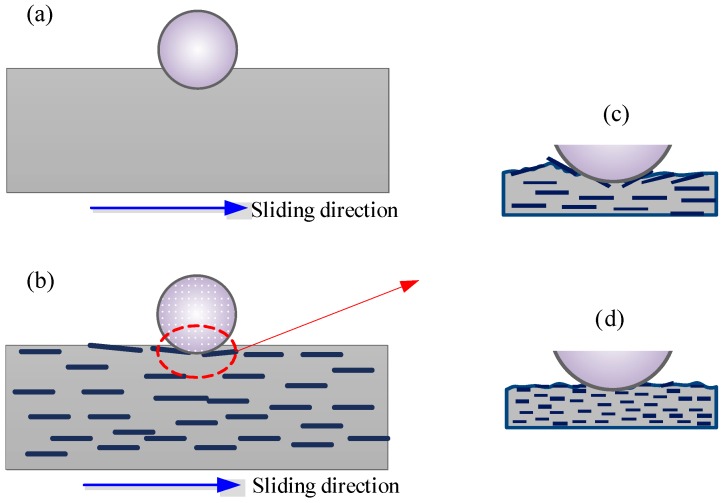
Schematic diagram of contact and wear of different NBR specimens.

The results of tribological tests indicated that the COF of NBR reduced with filler of micro glass flakes. This is because the contact surface changed with GF filler. Figure 12 shows the schematic view of contact and frictional process of NBR against steel ball. On one hand, it can be discovered from Figure 12a,b that the contact area and deformation of NBR decrease with GF-filled NBR. This is caused by the enhancement of hardness and load-carrying capacity with GF filler. On the other hand, the GF appeared at the friction interface during the sliding process. Accordingly, the real contact range at the interfaces between the NBR sample and steel ball decreased due to the GF filler. With regard to GF-B and GF-M, due to the contact normal pressure, rough steel ball surface and tangential force, the bigger GF becomes warped up and could fall off from the rubber surface as shown in Figure 10b–d. During the sliding process, the chips of GF appear on the rubber surface. This phenomenon leads to the reduction of the contact region between the NBR sample and steel ball. Thus, the adhesive force between the rubber and steel ball interfaces reduces with GF filler. As a result, the COF decreases after filled with GF. Moreover, the GF-S with the smaller size is not easily warped up compared with GF-B and GF-M in this study, which can be found in Figure 10d and Figure 12d. Thus, the NBR sample filled with GF-S shows a lower COF comparing to GF-B and GF-M samples.

### 4.2. Effect of GF Filler on the Wear Behavior of NBR

As mentioned above, Figure 8 displays the relationship of the wear rate with respect to the sliding time. It can be seen that *R_w_* is dependent on the sliding time. From Figure 8, we can easily observe that the *R_w_* decreases as the increase of time. It is because of the augment of contact region and the effect of rolling of debris on the contact interfaces during the successive wear process. As shown in Figure 10a, due to the repeated friction and contact process, the crack might appear when the elongation reaches a critical value, and the wear debris are crimping and rolling by the edge of contact during the consecutive friction process [35]. Afterwards, the detached rubber particles with the form of curled debris appear from the surface layer, when the crack is propagating to meet another. Furthermore, this outcome contributes to the production of curl pattern on the rubber surface and enhances morphology matching at the contact interfaces between the NBR and steel ball. Once curl-shaped worn pattern occurs, it will expand gradually under the condition of reciprocating pressure and drag during the sliding process. Finally, the curl-shaped debris are torn apart from the rubber matrix. Many researchers studied the relationship of wear morphology and the extension of crack [36]. The fatigue-fracture mechanism and decomposition caused by mechanochemistry could be used to explain the wear failure of rubber [35,40]. Gent and Pulford [41] noted that the occurrence of the oil grinding dust marks the occurrence of mechanochemistry decomposition. In this study, the transfer film is not observed, and the debris were dry. Therefore, the formation of curled debris is due to the adhesive wear and fatigue wear. This indicated that the wear mechanism of NBR is mainly adhesive and fatigue-fracture failure.

The original NBR shows a higher wear rate at the initial of sliding process, and then decreases with the increase in friction time. When the time reaches to 2 h, the wear rate tends to be stable as shown in Figure 8. Compared with the original NBR, the three GF-filled NBR specimens behave a better wear-resistant. Additionally, the wear loss shows a slight reduce with the decrease of GF size. This is ascribed to the reinforcement of the micro glass flakes. The GF-filled NBR samples behave a higher hardness compared to that of the unfilled sample which is indicated in Table 3. The wear surface patterns are different between the filled and unfilled NBR specimens, as shown in Figure 10. The degree of surface wear of GF-filled NBR became lower than that for the unfilled NBR. The typical curl-shaped patterns on the wear surface were observed as shown in the SEM images. For the GF-filled NBR specimens, the GF chips were obviously noticed on the wear surfaces of GF-B-, GF-M- and GF-S-filled NBR as shown in Figure 10b–d. From the schematic diagram (Figure 12c,d), it is indicated that the GF chips could improve the contact interface, which can prevent the extension of rubber curl-shaped patterns and reduce the crack at the surface due to the decrease of the stress concentration. Thus, the wear-resistant of NBR enhanced after mixed with GF fillers.

**Figure 13:**
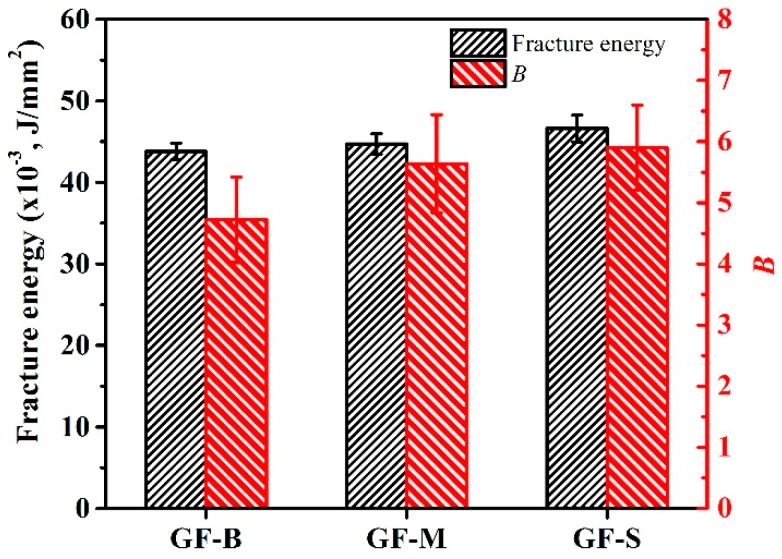
Fracture energy and semiempirical constant B of interfacial strength between glass flake and rubber matrix.

Additionally, the fracture energy can describe the energy change of extension of crack, which is a common application for analysis of the fatigue behaviors of polymers [42]. The average fracture energy is listed in Table 3. For each specimen, five tests are applied under the same condition. From Figure 13, it can been seen that the value changes of GF-B-, GF-M- and GF-S-filled NBR are slight. Generally, previous researches [43,44,45] indicated that the polymer fracture energy can be influenced by properties of filler, such as dispersity, volume fraction, morphology, interfacial strength, etc. In this study, the fillers’ morphologies and volume fractions are basically the same which can be found in Figure 1 and Table 1. Furthermore, from the fracture surfaces investigated by a low temperature brittle fracture method as shown in Figure 5, most of the GF fillers are horizontal in the rubber matrix after being cured by the hydraulic vulcanizing press process and the filler has a good dispersity for three GF fillers. Therefore, the fracture energy is majorly affected by the interfacial strength.

As previous study indicated, the Pukánszky equation is generally recognized to the filled elastomer materials [46]. The tensile strength of elastomer materials and the relationship of volume fraction to interfacial strength can be shown as follows:

(4) $$\sigma_{c}=\left(\frac{1-\nu_{f}}{1+2.5\nu_{f}}\right)\sigma_{m}\exp(B\nu_{f})$$

where *σ_c_* and *σ_m_* are the tensile strength of filled and unfilled polymer. *B* is semiempirical constant, and the higher value of *B* shows stronger interfacial strength at the interface of the filler and polymer. *v_f_* is the filler’s volume fraction. The semiempirical constant B of GF-filled NBR of GF-B, GF-M and GF-S are about 4.72, 5.64 and 5.90, respectively. As Equation (3) indicated, the interfacial strength between GF filler and rubber matrix behaves the same change in the fracture energy. Furthermore, the specific surface area increased with the decreasing of GF size. This leads to the enhancement of the interfacial strength at interface of the GF filler and rubber matrix. This is also contributing to the improvement of the interfacial strength and is beneficial for enhancing the wear resistance of NBR.

## 5. Conclusions

The nitrile rubbers filled with micro glass flake were prepared with different size fillers. Universal test machine, DSC and DMA were used to characterize the NBR specimens and the frictional and wear properties were investigated using a ball-on-flat mode tribometer.

The tribological test results showed that the COF reduced and the wear resistance improved for NBR filled with GF. The glass flake fillers appeared on the worn surface could restrain the present and extension of the curled patterns on the surface, which could improve the wear resistance property of rubber. However, the hard glass flake fall off from the rubber matrix would accelerate the wear of counterpart metal ball. Furthermore, the analysis of fracture surface manifested that the filler of micro glass flake possesses a good dispersity in the rubber matrix and good interfacial action. Moreover, filler with smaller size is more conducive to enhance the interfacial strength of the polymer matrix. This contributes to the improvement of the surface interaction and is beneficial for enhancing the wear resistance of NBR.

## Figures and Tables

**Table 1:** Chemical constituents of micro glass flake (‘C’ glass).

SiO_2_	K_2_O	B_2_O_3_	ZnO	Na_2_O	MgO	CaO	Al_2_O_3_	TiO_2_
64–70%	0–3%	3–8%	0–5%	11–18%	1–4%	3–7%	0–5%	0–3%

**Table 2:** Formula of nitrile rubber (NBR) vulcanizates reinforced with micro glass flake (GF).

Ingredient	NBR	Stearic Acid	ZnO	CBS	TMTD	Sulfur	GF
PHR	100	1	5	1.5	0.2	1.7	15

**Table 3:** Mechanical properties of GF-filled NBR vulcanizates.

Specimens	Unfilled NBR	GF-B	GF-M	GF-S
Elastic modulus (MPa)	0.63 ± 0.01	0.63 ± 0.01	0.65 ± 0.01	0.66 ± 0.02
Tensile strength (MPa)	6.24 ± 0.3	6.72 ± 0.3	7.07 ± 0.4	7.17 ± 0.3
Storage modulus (MPa, 20 °C)	12.29 ± 0.03	13.28 ± 0.02	13.35 ± 0.02	13.51 ± 0.02
Shore hardness (A)	44 ± 0.8	47 ± 1	49 ± 0.8	53 ± 1
Fracture energy (×10^−3^, J/mm^2^)	41.7 ± 1.1	43.8 ± 1	44.7 ± 1.2	46.6 ± 1.3
